# Risk factors for poor bereavement outcomes and the support needs of adult bereaved refugees within the psychosocial-cultural concept of personhood: a systematic review

**DOI:** 10.3389/fpubh.2025.1517237

**Published:** 2025-05-01

**Authors:** Çiğdem Fulya Dönmez, Muzeyyen Seckin, Cara Bailey

**Affiliations:** ^1^School of Medicine, Dentistry and Nursing, University of Glasgow, Glasgow, United Kingdom; ^2^Faculty of Health Science, Mugla Sitki Kocman University, Fethiye/Mugla, Türkiye; ^3^School of Nursing and Midwifery, Institute of Clinical Sciences, College of Medical and Dental Sciences, University of Birmingham, Birmingham, United Kingdom

**Keywords:** bereavement, refugee, risk factors, needs, personhood

## Abstract

**Background:**

The majority of refugees are exposed to trauma and multiple deaths and often face difficulties in coping with the death of someone close to them. This review aimed to systematically synthesize evidence regarding the risk factors associated with poor bereavement outcomes and the support needs of refugees during bereavement, using the ring theory of personhood as a guiding framework to inform service provision and policy development.

**Methods:**

A systematic review was conducted using five citation databases (PsycINFO, CINAHL, SocINDEX, MEDLINE, and Web of Science), along with gray literature sources, to explore the bereavement support needs of refugees worldwide. The review followed the ring theory of personhood framework. A standardized data extraction tool (34) was used to analyse studies that included adult refugees who had experienced the death of a family member/friend. Two reviewers independently assessed the quality of the included studies to reduce bias. Due to the heterogeneity across the included studies, a narrative synthesis was conducted independently by two reviewers, with consensus reached on the final analysis.

**Results:**

A total of 11 studies (eight quantitative, two mixed-methods, and one qualitative) were eligible for inclusion, representing a combined sample of 3,053 refugees. Across the rings of personhood, various risk factors related to bereavement were identified, including female sex, age, comorbidities, cultural and religious beliefs/values, and social factors. The findings indicate that refugees would benefit from culturally sensitive diagnostic tools for the early detection of grief symptoms, along with psycho-socio-cultural bereavement support interventions. Such interventions could enhance healthcare providers’ understanding of refugees’ perspectives, values, and preferences, thereby improving the quality of care provided.

**Conclusion:**

This review identified a wide range of culturally sensitive assessments and support needs for bereaved refugees. A personalized approach to bereavement assessment and care is recommended to facilitate a more comprehensive and existential understanding of bereavement and grief in research, clinical practice, policymaking, and community-based support systems. Further research is warranted to examine the long-term effects of bereavement on refugees’ mental health and to explore strategies for providing sustained support throughout the refugee journey.

**Systematic review registration:**

https://www.crd.york.ac.uk/PROSPERO/view/CRD42022370126, Identifier CRD42022370126.

## 1 Introduction

The increasing number of refugees in recent years poses a public health concern, as many experience significant health issues and struggle to access healthcare. Between 2000 and 2023, more than 100 million people were forced to leave their homes due to violence, conflict, persecution, or events severely disrupting public order (1). Overall, 52% of all refugees and other individuals in need of international protection have come from just three countries: Syria (6.5 million), Afghanistan (6.1 million), and Ukraine (5.9 million) (1). Türkiye and Iran each hosted 3.4 million refugees, representing the largest populations globally. Germany ranks third with 2.5 million, followed by Colombia with slightly fewer than 2.5 million, and Pakistan, which hosts 2.1 million refugees (1). The unprecedented rise in displacement worldwide exposes refugees to widespread violence, trauma, and instability while also exacerbating mental health problems. Refugees often face a range of psychosocial challenges due to their displacement, including exposure to violence, loss, and uncertainty (2).

Bereavement is a critical public health issue with profound social and psychological consequences for individuals and societies (3). In the general population, grief is a natural response to loss, but it can lead to significant mental health problems such as depression, anxiety, and prolonged grief disorder, particularly in the absence of adequate social and emotional support (3, 4). Compared to the general population, refugees have a heightened risk of mental health disorders such as depression, post-traumatic stress disorder, and prolonged grief disorder (PGD) (5). The experiences of the migration journey contribute to bereavement risk factors, and individual (e.g., age, gender), family (e.g., relationship to the deceased), and macro-level factors (e.g., lack of access to health services or political and administrative issues related to migration) lead to poor bereavement outcomes (e.g., high intensity of grief and prolonged bereavement) (2, 5). The majority of refugees are exposed to trauma and multiple deaths and experience difficulties adjusting to the death of loved ones (2, 6–15). A recent narrative systematic review (2) highlighted that the prevalence of prolonged grief disorder among refugees and immigrants was 34%, with older refugees at higher risk of experiencing traumatic and multiple deaths of first-degree relatives. Prolonged grief disorder is a distinct syndrome characterized by severe grief reactions for an extended period of time (a minimum of 12 months) following the death of a significant person (11, 14, 16) and has recently been recognized in the International Classification of Diseases (17). A narrative review (18) found that 32% of bereaved refugees scored ‘poor’ in bereavement outcome ratings, significantly higher than prevalence studies in general population samples (19). Similarly, a recent systematic review with meta-analysis reported rates of prolonged grief disorder as 9.8% in the general adult population, demonstrating the severity of bereavement outcomes for refugees.

While poorer bereavement outcomes related to experiences of refugees, including bereavement arising from war, torture, detention, or during the process of fleeing persecution, have been documented, evidence regarding bereavement care needs, including risk factors from the psycho-socio-cultural perspective, is limited (7, 18). This systematic review aligns with the World Health Organization’s remit (20) to address an unmet global health need by tackling evidence-practice gaps in bereavement care. It aims to contribute to dignified, person-centered care by supporting individuals at heightened risk of poor bereavement outcomes, particularly during times of mass loss. By synthesizing evidence on the bereavement support needs of refugees, the review addresses critical gaps in the literature, focusing on the psycho-socio-cultural concept of personhood—a dynamic process where individuals navigate and participate in a cultural system of meanings as both subjects and social actors. Additionally, the notion of “personhood” is inherently part of person-centeredness, including key cultural values, norms, and ideas characterizing the person, that is, an understanding of what a person (21).

Learning more about the personhood of bereaved refugees in the context of their support needs is vital for providing dignified, person-centered bereavement care and developing a personalized support system. This review will support the development of evidence-based policies and practices that involve culturally adapted and more comprehensive bereavement interventions for refugees from diverse backgrounds. Findings from this systematic review can also facilitate the construction of a standardized tool based on the psycho-socio-cultural concept of personhood for use by healthcare professionals. Furthermore, this review may contribute to the establishment and improvement of support programs for prolonged grief disorder, providing a comprehensive, holistic perspective.

### 1.1 Theoretical lens

**Figure 1:**
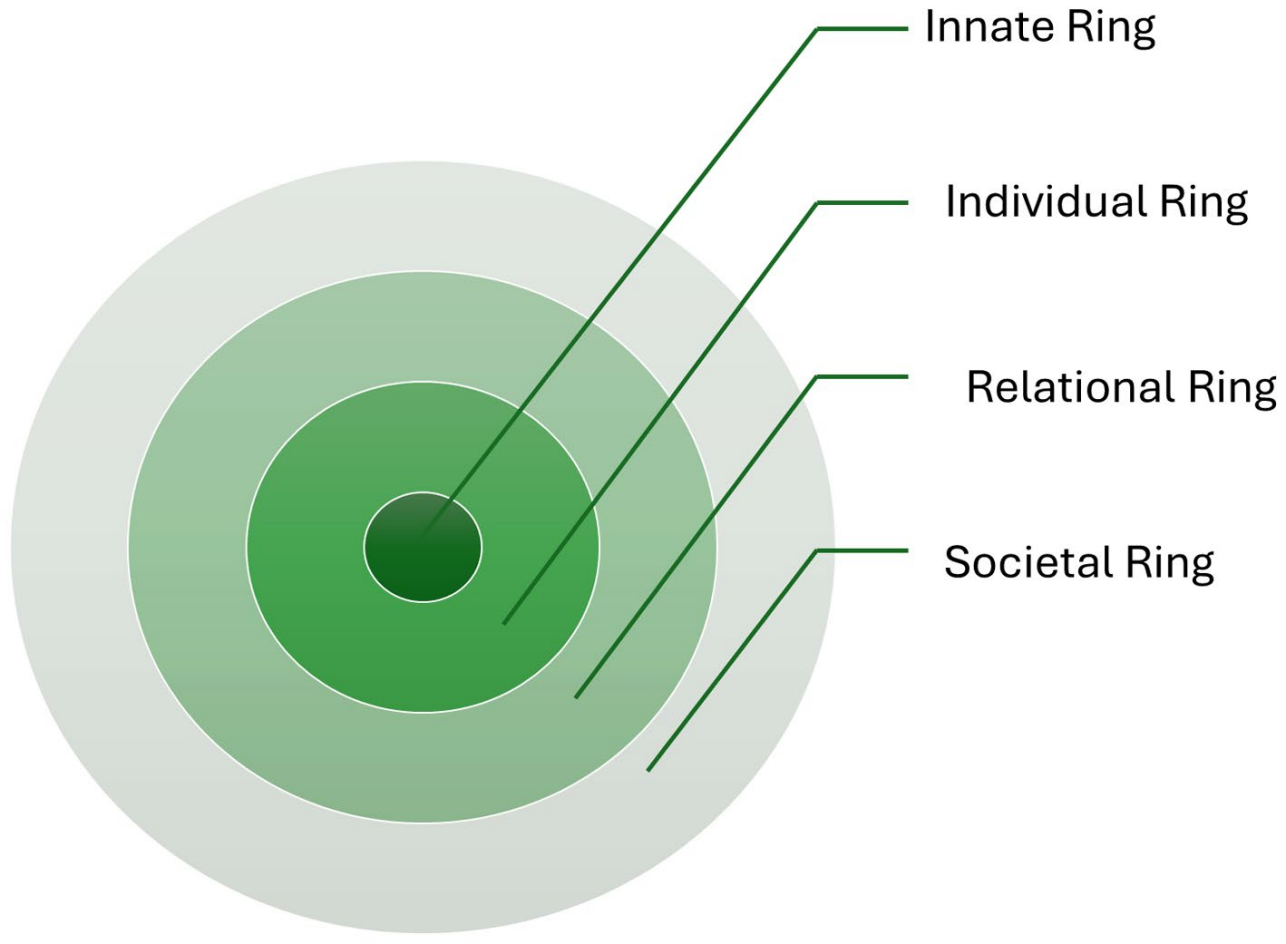
Ring Theory of Personhood. Radha Krishna’s Ring Theory of Personhood (23).

As the concept of the psycho-socio-cultural notion of personhood, or “what makes you, you” is influenced by prevailing religious beliefs, experiences, societal mores, and moral and cultural codes, the current study of dignified and person-centered bereavement care for refugees requires a holistic and longitudinal evaluation. For that comprehensive evaluation, we have adopted Krishna and Alsuwaigh’s Ring Theory of Personhood (21–25), defined in terms of four domains, including the Innate, Individual, Relational, and Societal Rings (Figure 1).

The Innate Ring encompasses an individual’s genetic and historical characteristics, including ethnicity, age, gender, society, personality, and country. The Individual Ring emphasizes the significance of an individual’s unique values, beliefs, roles, traits, preferences, mores, norms, and principles. The relational ring involves close personal relationships that are important to the individual, such as dear friends and family members. The Societal Ring comprises less significant and more impersonal relationships, including acquaintances and colleagues. This ring also includes societal obligations, expectations, roles, professional standards, and laws that govern conduct within one’s society (21–26).

This clinically evidenced holistic concept of personhood has been created within palliative care settings. Recent studies based on the Ring Theory (22–26) suggest that the psycho-socio-cultural characteristics of personhood, influenced by prevailing religious beliefs, societal mores, and moral and cultural codes, for people needing palliative care, may be mapped using the Ring Theory of Personhood to provide person-centered and dignified end-of-life care. Personhood in bereavement reflects an individual’s unique identity, autonomy, and relational existence, recognizing that grief is both personal and shaped by social and cultural connections (27). It acknowledges the capacity to experience loss, make meaning of it, and adapt while also highlighting the role of relationships, social support, and cultural practices in shaping the grieving process. Therefore, the Ring Theory of Personhood has been adopted to provide dignified and person-centered bereavement care for bereaved refugees from diverse backgrounds in this rapid review. Using this tool may help develop evidence-based practices that involve a cultural component specific to bereaved refugees from various backgrounds. This may also help healthcare professionals respond more appropriately and sensitively to individuals’ needs since they have better-informed insights into the person.

### 1.2 Aims and research questions

This systematic literature review primarily aims to systematically synthesize the evidence on the risk factors for poor bereavement outcomes and the support needs of bereaved refugees based on ring theory to inform service provision and policy. These aims help provide evidence-based recommendations and resources for the implementation of bereavement support for refugees who have experienced the death of a family member or friend.

A research agenda was built around this systematic review, centered on two main research questions: (i) How can the risk factors for poor bereavement outcomes be explained using ring theory? (ii) What are the needs of bereaved refugees in the context of the psycho-socio-cultural concept of personhood to inform service provision and policy?

## 2 Materials and methods

### 2.1 Design

For the purposes of this research, our team undertook a systematic review, an approach to evidence synthesis that produces findings in a shortened time frame and provides a broad overview, as opposed to an in-depth critique of the evidence available on a specific topic (28). We selected this method to deliver evidence that might rapidly guide the design of bereavement support systems for refugees. The review was registered with Prospero (CRD42022370126) and is reported following the Preferred Reporting Items for Systematic Reviews and Meta-Analysis guidelines (29) (see Supplementary File S1 for PRISMA checklists).

### 2.2 Inclusion/exclusion criteria

The inclusion criteria were as follows: reports of qualitative, quantitative, and mixed-methods original research studies; adult refugees who had experienced the death of a family member or friend; and studies published in the English language. We also included studies with no restrictions on publication date to gather all the necessary data.

We excluded reports published in non-peer-reviewed journals, as well as commentaries, editorials, case studies, conference abstracts, and books. Further details of the inclusion/exclusion criteria are presented in Supplementary File S2.

### 2.3 Search strategy

We searched the following databases from inception to 2nd December 2023: PsycINFO, CINAHL, SocINDEX, MEDLINE, and Web of Science (see Supplementary File S3 for search strategy). We explored gray literature to access articles not published by commercial publishers through Open Gray (30), Google, and Google Scholar. Gray literature was included only if it met predefined quality criteria, ensuring relevance and credibility, while non-peer-reviewed sources that did not meet these standards were excluded. The searches were repeated to capture data up to 13th June 2024, based on a preregistered protocol to ensure transparency. In addition, we conducted forward and backwards citation tracing, reviewing studies that cited the included studies and screening the reference lists of those studies.

### 2.4 Screening process

**Figure 2:**
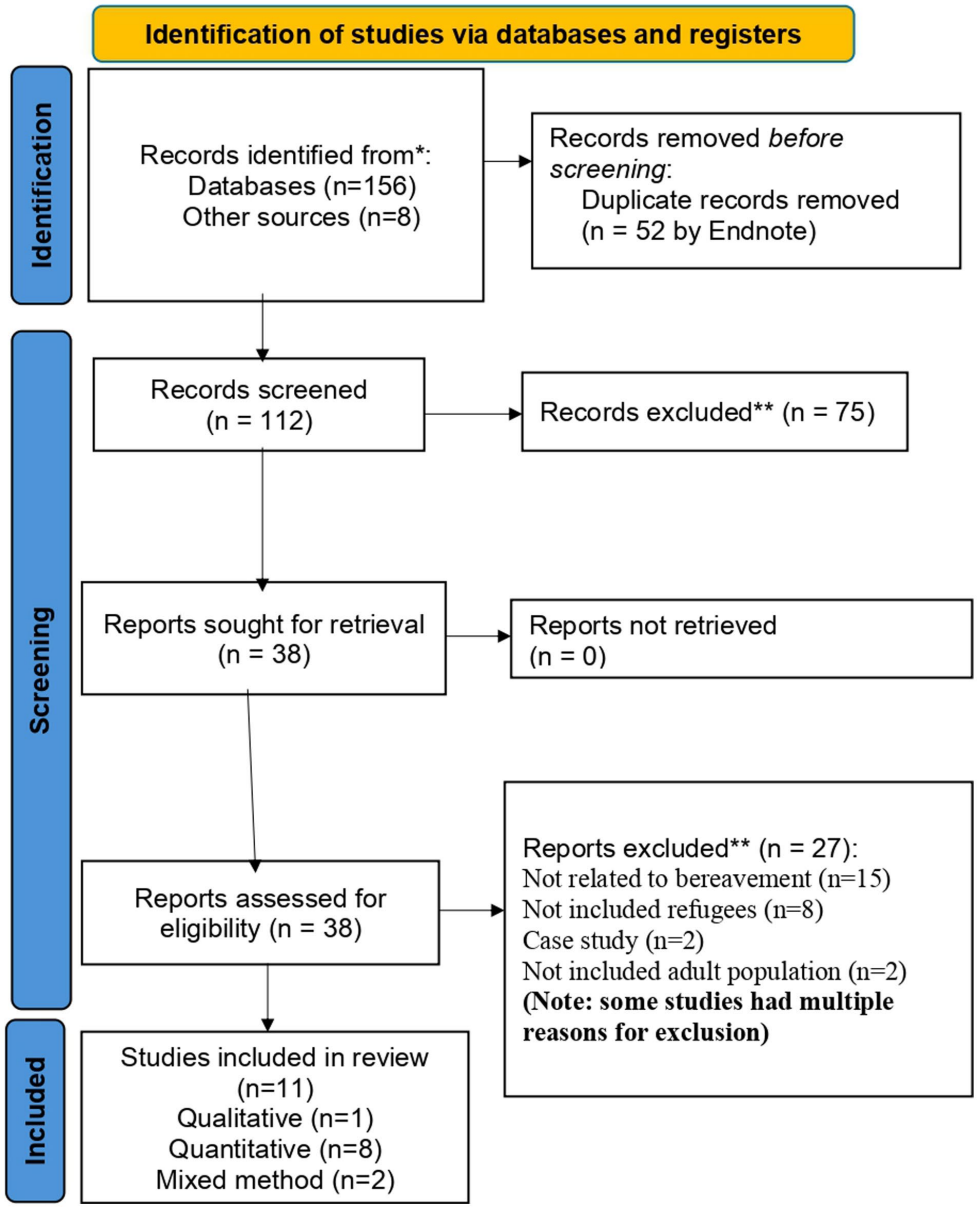
Preferred reporting items for systematic reviews and meta-analysis flowchart to identify reviewed and included articles.

Citations were managed using EndNote X9 software (31). Duplicates and unrelated titles were removed. Title and abstract screening were conducted by the first author, Ç.F.D., and the second author, M.S. Records that satisfied the inclusion criteria were retrieved and fully read. Full texts were double-screened by Ç.F.D. and M.S. Any discrepancies regarding whether a study met the inclusion criteria were resolved through discussion with the other reviewing author. There were no instances where agreement was not reached. The screening and selection of articles and guidelines were documented following the PRISMA flow diagram (Figure 2).

### 2.5 Data extraction

We used a standardized data extraction tool for the included studies in accordance with systematic review guidance (32). Two reviewers (C.F.D. and M.S.) independently conducted the data extraction, and any disagreements were resolved by consensus through a review of the data and clarification of discrepancies until a mutual agreement was reached. The data extraction tool, developed by C.F.D. and M.S., captured the following information: the author/year, the country, the study title, the design/methodology/method of data collection, the sample/population, the aim, bereavement support needs, and the domains of the Ring Theory of Personhood.

Articles were screened by matching keywords and titles related to the bereavement needs of refugees. The initial search retrieved 164 articles from different databases and sources; of these, duplicates (*n* = 52) and unrelated titles (*n* = 75) were removed. The remaining 38 articles were screened, and 27 were excluded, leaving 11 articles for the final review (Figure 2).

### 2.6 Data analysis

Due to the heterogeneity of the included studies, we selected narrative synthesis (28) as the best method for analyzing them. Following Popay et al. (33), our synthesis involved four steps. The first step is developing a ‘theory of change’ (the theory underpinning this review is that a clear understanding of the components of bereavement support for refugees is needed to build a rigorous empirical base). The second step is developing a preliminary synthesis of the findings from the included studies. The third step is exploring relationships within and between studies, and the final step is assessing the strength of the evidence, which results in a summary of the knowledge related to the specific review question to inform future practice and policy. In our review, we extracted and elaborated on the overarching themes from the different studies using an inductive approach without a previously established categorization. We discussed, refined, and structured the emerging findings according to the review question and aims. Additionally, Microsoft Excel was utilized to compute basic descriptive statistics, including the total number of review participants, the percentages of females and males, and the percentage of individuals with prolonged grief disorder.

### 2.7 Quality assessment

Two reviewers (Ç.F.D and M.S.) independently assessed the quality of the included original study reports, resolving any discrepancies through discussion. The independent scores in the quality assessment were calculated by Ç.F.D. and M.S. and then compared for each study. Discrepancies were discussed before reaching a final score decision. We used the appraisal tool developed by Hawker et al. (34) to appraise qualitative, quantitative, and mixed methods studies. This tool uses a checklist to assess study quality and calculate a summed score [ranging from 40 (‘good’) to 10 (‘very poor’)]. It provides a comprehensive framework for assessing the quality of both quantitative and qualitative studies in systematic reviews. This tool allows for a structured evaluation of methodological rigor, helping to ensure that the studies included in the review are robust and reliable, thus strengthening the overall conclusions.

The quality of the majority of the studies (*n* = 9) was ‘good’, with an average score of 34 out of 40 points across the 11 included studies. Points were assigned to each item as follows: good = 4, fair = 3, poor = 2, very poor = 1. The quality of the two studies was ‘fair’. All studies provided a clear abstract (range 3–4), a detailed introduction with an appropriate aim (all scores = 4), and sufficient information regarding the method of research (range 3–4) and data analysis (range 3–4), along with clear results (range 3–4) and more sufficient implications and usefulness of the findings (range 3–4). However, some scores on the items of ethics and bias were low (‘very poor’ or ‘poor’). Additionally, a few studies had unclear information concerning sampling techniques/procedures and the transferability and generalisability of the results (range 2–4). During the quality assessment, one study was excluded due to poor quality (35). A summary of the methodological quality of the included studies is presented in Supplementary File S4.

## 3 Results

### 3.1 Demographics

All of the 11 included studies were published between 2008 and 2021 and came from the following countries: Austria (*n* = 5), Germany (*n* = 2), the United States (*n* = 2), France (*n* = 1), and Switzerland (*n* = 1). The study designs were primarily quantitative (*n* = 8). Two studies used mixed methods, and one study was an exploratory qualitative study. The total number of adult participants was 3,053 (range 10–1,245), including 45% women (*n* = 1,379) and 55% men (*n* = 1,674) from the included studies (Table 1). The mean age in the included studies ranged from 29 to 54 years. A diverse refugee population from various countries (Africa, Afghanistan, Bhutan, Bosnia, Eritrea, Iran, Iraq, Somalia, Ethiopia, Myanmar, Libya, Syria, Egypt, Pakistan, Sri Lanka, India, and West Papua) was exposed to violence-related trauma and the close family death in the included studies (see more in Supplementary File S5). In the articles, the main issues related to bereavement needs among refugees were extracted for narrative synthesis.

**Table 1:** Details of included studies.

Details of included studies		
		Total (*n* = 11)
Summarized article characteristics. (*n*) represents the number of studies with the characteristic		
Country^#^ (in which the study was conducted)	Australia, *n* (%)	5 (45)
	Germany, *n* (%)	2 (18)
	United States of America, *n* (%)	2 (18)
	France, *n* (%)	1 (9)
	Switzerland, *n* (%)	1 (9)
Date of publication	2008–2015, *n* (%)	3 (27)
	2015–2021, *n* (%)	8 (72)
Study design	Survey, *n* (%)	6 (54)
	Mixed-method, *n* (%)	2 (18)
	Prospective cohort, *n* (%)	2 (18)
	Cross-sectional, *n* (%)	1 (9)
	Exploratory qualitative, *n* (%)	1 (9)
Sample size range	<100 participants, *n* (%)	4 (36)
	100–500 participants, *n* (%)	6 (54)
	>500 participants, *n* (%)	1 (9)
Summarized patients’ descriptions were included in 11 articles. (*n*) represented the number of participants		
Sample size	Total number of patients	3,053
	Male, *n* (%)	1,674 (54.8)
	Female, *n* (%)	1,379 (45.1)
Mean age range*	29–35 years old, *n* (%)	4 (36)
	36–50 years old, *n* (%)	4 (36)
	>51 years old, *n* (%)	1 (9)

*Two studies did not report mean age. ^#^Counrty in which the study conducted.

### 3.2 Findings based on the domains of the ring theory of personhood

The concept of personhood includes four main domains: the innate, the individual, the relational, and the societal. The findings of the included studies were mapped onto these domains (Table 2).

**Table 2:** Risk factors for poor bereavement outcomes and the support needs of bereaved refugees.

Risk factors according to the ring theory of personhood	Bereavement support needs
Innate ring Female sex (36–39) Older/advanced age (36–38) Religious belief Female and older individuals with multiple traumatic events or deaths in the context of the loss of culture and support (13) Comorbidity and other medical conditions such as depression, anxiety, and disability (12, 13, 36, 38, 40, 41)	Better diagnostics (e.g., culturally sensitive) tools for early grief symptoms detection (12, 13, 40) Culture-adopted grief-focused interventions (12, 13, 36, 38, 40) The utility of diagnostic criteria of prolonged criteria (41) Medical examination upon their arrival (38, 40) Better mental health and culturally sensitive assessment tools (39) Bereavement rituals support (42) Psycho-cultural support (42) Maintaining cultural, traditional, and religious belief support (12, 13) Family support (37, 39, 43) Social (42) and community support (37, 39, 43) Psychological support with increased counseling (13) and mental health services access (36, 38) Grief-focused interventions/strategies (13, 38, 40) Alternatives for mental healthcare due to mental health stigma (39)
Individual ring Cultural beliefs and values (9, 39, 41) Not being able to join bereavement rituals (39, 42) Traumatic event: the number of traumatic events (12) and deaths (9) Individual beliefs regarding the deaths of close ones (42)	
Relational ring Multiple traumatic events and deaths, including the first-degree relatives (40, 42) Being eldest sibling (42) Having children (12) Being not married (36) Uncertainty about close one (39)	
Societal ring Difficulties in social and professional integration in the new countries (39, 42) Social integration difficulties such as social isolation and bad living conditions (36, 40) Loss of their connection with close ones and social networks (39) Political and administrative issues related to migration (39, 40, 42) Longer immigration duration (9, 42) Having trust issues with the host country (36) Feeling discriminated against in the society (36) Economic and financial issues (42) Unemployment (36, 40) Uncertainty about residential status (39) The lack of health service access (39)	

#### 3.2.1 The innate ring

Individual characteristics influence the severity of bereavement and grief-related issues. Despite mixed evidence in the studies, females (36–39) and older individuals (36–38) were at a higher risk for severe grief and prolonged grief disorder. In particular, those with multiple traumas and traumatic deaths experienced prolonged grief disorder in association with the loss of culture and support (13). Females also appear more emotionally able to express their feelings compared to men in some cultures, which poses a risk for severe grief (39). Having additional comorbid or other clinical conditions, such as mental distress, depression, somatic complaints, post-traumatic stress disorder, anxiety, and disability, was significantly associated with prolonged grief disorders among refugees (12, 13, 36, 38, 40, 41).

#### 3.2.2 The individual ring

An individual’s beliefs, values, and views influence the level of bereavement complications and symptoms. Cultural beliefs such as rebirth/reborn and *khyâl* attack (a cultural explanatory model of somatic symptoms) are associated with fewer grief complications or prolonged grief disorder (41). Additionally, attending bereavement rituals for saying a final farewell helps to decrease the degree of grief experienced (39). Refugees reported feeling guilty, dazed, lonely, and experiencing bitterness and emptiness when they did not attend bereavement rituals. The lack of rituals limited their ability to accept the death of someone close, which in turn increased feelings of guilt and despondency (42). Beyond culture, historical and contextual factors contributed to the content and configuration of grief reactions regarding one’s expression and emphasis (9, 39). Refugees with traumatic death experiences, including war-related fatalities, had a higher prediction of prolonged grief disorder. There was an association between the number of traumatic events (12) or losses (9) and higher levels of grief symptoms. If they face deaths expected in old age or after a long-term clinical condition, they experience fewer grief complications (42).

#### 3.2.3 The relational ring

The severity of prolonged grief disorder was associated with experiencing multiple traumatic deaths involving first-degree relatives (40, 42). Eldest siblings faced severe grief complications (42). Refugees who are the eldest siblings, married, or in a common-law union are at higher risk for prolonged grief disorder due to the weight of their hierarchy and others’ expectations of them. Another study identified that married refugees reported less prolonged grief disorder (36). Refugees with children are also at higher risk for prolonged grief disorder (12). Additionally, uncertainty about what happened to their loved ones increased the severity of grief (39).

#### 3.2.4 The societal ring

Refugees’ previous traumatic experiences (e.g., war-related, detention, abuse, or postmigration living difficulties) affected their social relationships. They have faced greater challenges in achieving social and professional integration (39, 42). These difficulties (e.g., social isolation and poor living conditions (40)) were associated with increased prolonged grief disorders and grief symptoms (36). The loss of connection with culture, homeland, and their close relationships or social networks worsens grief (39).

Political, economic, and administrative issues within the context of migration negatively affected refugees’ bereavement experiences (39, 40, 42). Refugees with longer immigration durations reported more problems related to their social lives (42), increased prolonged grief disorder associated with their unemployment status (36, 40), and overall complicated bereavement symptoms (9). Those who faced the death of a close one in their native country were unable to participate in their bereavement rituals due to administrative and financial problems (42). Furthermore, they experienced higher levels of prolonged grief disorder when they encountered trust issues and discrimination in host countries (36). Uncertainty about residential status and lack of access to health services posed risks for grief-related issues among refugees (39).

### 3.3 Bereavement support needs

Unsurprisingly, there was a strong association between refugees’ previous traumatic experiences and bereavement/grief difficulties, as these experiences shaped their perception and ability to cope with adapting to a new life and accepting the death of a loved one (Table 2). Culture-adopted grief-focused interventions and therapies may be key components of bereavement care (12, 13, 36, 38, 40). Cultural values and beliefs deeply influence grief responses among refugees. There is a critical need to support the maintenance of cultural traditions, religious beliefs (12, 13, 41), and bereavement rituals (42). Additionally, there is a need for better diagnostic tools for early grief symptom detection (12, 13, 40), which can be utilized during medical examinations upon their arrival (38, 40). Describing the psychological context of refugees with better mental health (39), along with cultural, religious, and historical considerations, should be taken into account by healthcare providers to utilize the diagnostic criteria for prolonged grief disorder (41). Culturally sensitive assessment is important, as studies have shown that there are cross-cultural differences in grief symptoms, which are essential for accurately understanding and addressing the unique ways individuals from diverse cultural backgrounds express and experience grief (39, 41). Furthermore, grief-focused comprehensive psychological support may benefit this specific population (9, 42, 43). Considering the interaction between mental distress and grief-related problems in refugees, psychological support, along with increased counseling services (13) and greater access to mental health services, may be required for them (36, 38). However, in some cultures, seeking mental health consultation and support can carry a stigma (such as being labeled as ‘crazy’) (39). Therefore, alternatives for mental healthcare must be considered for these individuals (39). In this regard, social support (42) and family and community support (37, 39, 43) are important to reduce grief-related problems among refugees.

## 4 Discussion

**Figure 3:**
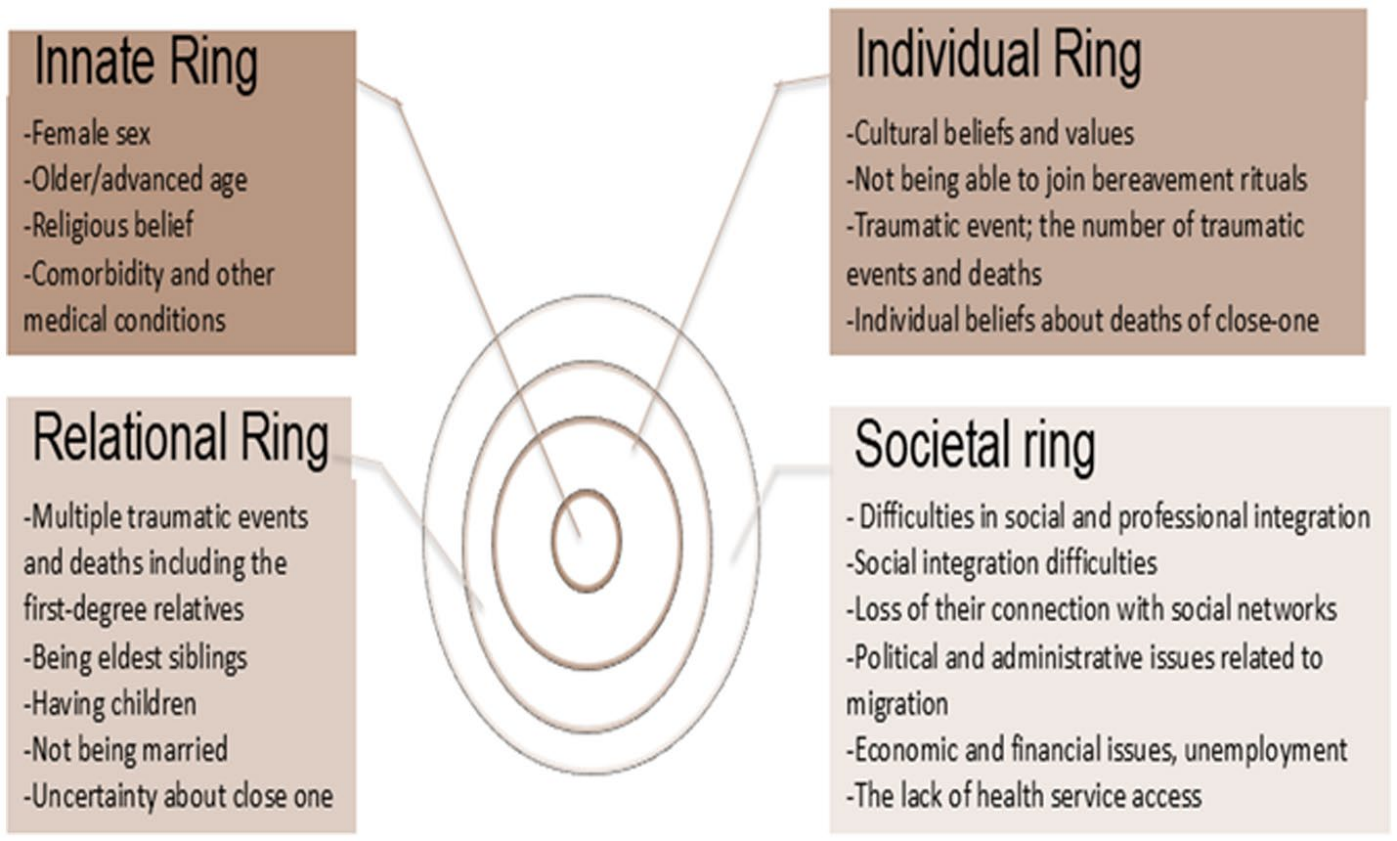
Findings on the rings of the theory.

To the best of our knowledge, this is the first review to systematically synthesize the evidence on the risk factors for poor bereavement outcomes and the support needs of bereaved refugees within the psycho-socio-cultural concept of personhood. By identifying how refugees’ bereavement is influenced by their previous traumatic experiences and close-on death experiences, this rapid review highlights the advantage of using the Ring Theory of Personhood as a broader framework to analyse the risk factors associated with bereavement-related issues among bereaved refugees (Figure 3).

Individual characteristics (e.g., biological sex, age, cultural and religious beliefs and values, and relationships) described in this review cause poorer bereavement outcomes. Consistent with previous literature, sociodemographic factors (such as age, sex, and education level) (2, 44) and trauma-related experiences (2, 18, 45–47) were highlighted as risk factors for bereavement. For example, female sex and older age are linked to a greater risk of experiencing grief-related issues. Female refugees and older individuals who experienced multiple traumatic events or deaths, along with the loss of cultural and social support networks, faced an increased risk of developing prolonged grief. In some cultures, there exists a sex-based vulnerability; in many societies, women are often encouraged to express their emotions openly, while men may face judgment for showing their bereavement emotions. This may explain the visibility of bereavement symptoms and outcomes for women. Additionally, due to advanced age, older individuals may face multiple losses simultaneously, compounding their bereavement outcomes. Individuals who have endured multiple traumatic deaths and losses are particularly vulnerable to poor bereavement outcomes and prolonged grief. One’s pre- and post-immigration experiences are associated with their mental distress and bereavement (2, 45, 48, 49). Mental health assessment of refugees is a core aspect of bereavement care (50), as it directly impacts public health by identifying those at risk of long-term psychological issues (5). Early intervention can mitigate the broader societal burden by preventing the escalation of mental health disorders, which can affect both individual wellbeing and community stability.

In previous literature, cultural and religious aspects (18, 45, 47) were highlighted as risk factors for bereavement. Cultural beliefs and practices influence individuals’ experiences and coping mechanisms with grief. In particular, participating in culturally significant bereavement rituals is crucial for many refugees. These rituals offer a sense of closure and community support, which can help alleviate feelings of isolation and despair. However, the inability to partake in these rituals due to displacement or financial constraints can exacerbate feelings of guilt, loneliness, and bitterness, hindering the acceptance of loss. The importance of cultural assessment (2, 18, 45–47, 50, 51) is highlighted to address bereavement for refugees. Without culturally assessing the bereavement experiences of refugees, healthcare providers cannot clarify expectations and the types of help needed (45). A clinical exploration of the cultural aspects of bereavement and grief among refugees upon their arrival is necessary through person-centered and open-ended questions (45, 51).

Refugees face unique challenges that exacerbate the severity of their grief and affect bereavement outcomes in their societies (2, 47). Social factors are also highlighted as risk factors for bereavement in this review. Guo, Alajarmeh (52) In this study, we conducted interviews with refugees in Jordan who were living with advanced cancer, as well as their caregivers. This research identifies the impact of fractured families and social networks, alongside compounded trauma, on refugees with advanced cancer and their caregivers. Compounded trauma is understood as the result of various historical, social, and political constraints that shape their personal experiences as refugees (53). Many refugees shared specific traumatic experiences, such as being forced to flee their home countries to escape severe human rights violations and enduring long-term physical and emotional suffering, including exposure to war, violence, and abuse (52). Seeking safety separated them from their family and social networks, which they would typically rely on to cope with the anxiety and stress of their cancer. Financial challenges have also become a major concern for refugees, particularly those with chronic illnesses, as their care is often not covered by host countries (52). Social factors, such as unemployment status, lack of access to mental healthcare services, mental health stigma, and feelings of discrimination, should be considered by healthcare providers and policymakers in these countries (48). To address these challenges, it is essential to provide refugees with education and support programs, ensuring they receive comprehensive assessments and care, have timely access to necessary services, and are given clear and accessible information to help them make informed decisions about their care and support (52).

There is an interaction between rings of personhood within the psychosocial and cultural context of the individuals. In this review, various interconnected factors have been identified as risk factors for increased bereavement-related issues or poor bereavement outcomes among bereaved refugees according to rings of personhood. Based on these findings, we provide evidence-based recommendations for bereavement support in refugees (Table 3). One conclusion from this review is that there is a need for comprehensive bereavement assessment and strategies for refugees to support their diverse cultural and religious backgrounds. This review outlines the lack of culturally sensitive and comprehensive psychosocial assessment tools and interventions. Culturally sensitive assessment is important because studies have found cross-cultural variations in grief symptoms (39) and different prevalence rates of prolonged grief disorder across cultures (54, 55). Such issues contribute to the ongoing difficulties related to bereavement care and prolonged grief disorder in this specific group. Therefore, there is a need for culturally sensitive bereavement-focused assessment and therapies that include families and communities while implementing aspects of personhood (2, 18). Although culturally sensitive interventions are crucial for prolonged grief disorder, findings from a scoping review indicate that seven out of 18 studies explicitly mentioned an approach to cultural adaptation for their targeted sociocultural groups, while 11 studies did not explicitly reference a specific intervention as the foundation for their culturally adapted approach (56). In a multicultural context, bereavement assessment tools and interventions must be adaptable to accommodate diverse cultural beliefs, practices, and grieving rituals. Culturally sensitive approaches may involve incorporating language-specific resources, illustrations, and respect for traditions, as well as considering social, cultural, environmental, and psychological factors that influence health behaviors to ensure interventions resonate with the unique needs of different cultural groups (56, 57). For example, the cultural adaptation of an Internet-based self-help app, following the Reporting Cultural Adaptation in Psychological Trials criteria, can enhance treatment adherence, acceptance, and motivation among grieving refugees (58).

**Table 3:** Evidence-based recommendations for bereavement support for refugees.

Evidence-based recommendations
The clinical exploration of cultural aspects of bereavement and grief among refugees upon their arrival is needed.
Healthcare providers should recognize that women and older people are at risk for poor outcomes of bereavement and provide person-centered care to this vulnerable group.
To eliminate the risk factors, the establishment and improvement of a dignified, personalized support system for the bereavement care of refugees by providing healthcare professionals with a comprehensive and holistic perspective should be provided.
Bereaved refugees need better culturally sensitive diagnostic tools for early grief symptoms detection and psycho-socio-cultural bereavement support interventions/strategies, which may increase healthcare professionals’ insight into refugees’ perspectives, values and preferences.
Healthcare providers and policymakers must consider relations between psycho-socio-cultural risk factors (e.g., lack of access to mental health services, mental health stigma and experiences of discrimination) with poor bereavement outcomes.
More empirical attention should be needed on the early detection of (psycho-socio-cultural and economic) risk factors of poor bereavement outcomes among newly resettled refugees in the host countries.
More evidence-based policies and practices are needed that involve culturally sensitive and more comprehensive bereavement interventions/strategies for bereaved refugees from a diversity of backgrounds.

In the context of the psycho-socio-cultural concept of personhood, a close examination of the risk factors for poor bereavement outcomes and the support needs of bereaved adult refugees is crucial to understanding the individual behind the bereaved refugee and providing more dignified, person-centered bereavement care. Regarding the findings of this review, further studies are needed to investigate the psycho-socio-cultural aspects of personhood related to the risk factors for poor bereavement outcomes and the bereavement support needs of refugees. More empirical attention is necessary for the early detection of psycho-socio-cultural and economic risk factors impacting poor bereavement outcomes among newly resettled refugees in host countries. The results of this review can provide important insights into the cultural processes shaping bereavement responses to healthcare services, assisting nurses in responding more appropriately and sensitively to individuals’ needs. Existing bereavement treatments, such as grief counseling, psychological support interventions, and family-based trauma interventions (56, 59, 60), can be recommended; however, adaptations may be needed to address the unique challenges faced by bereaved refugees, including cultural sensitivity, language barriers, and trauma-informed care. For example, integrative adaptation therapy may help reduce symptoms of persistent complex bereavement Disorder among refugees, as its ability to adapt to the psychosocial disruptions associated with the refugee experience potentially influences the course of complicated grief reactions (60). Displacement contributes to additional trauma and leads to poor bereavement outcomes for refugees, highlighting the urgent need for healthcare providers to develop the knowledge and skills necessary to address individuals’ needs effectively, alongside broader humanitarian and political issues (52). Adopting a public health approach is essential for developing comprehensive solutions to these challenges.

### 4.1 Strengths and limitations

This review has a few limitations. The risk factors of bereavement among bereaved refugees were generally based on survey studies from a limited number of countries, which restricts the generalizability of the results to broader populations or different countries, particularly other refugee populations. Studies typically assessed only a few sociodemographic factors (mainly biological sex and age) related to bereavement. Other factors might be linked to increased bereavement-related issues among refugees. English-based literature was utilized for this review; therefore, studies in other languages were excluded, limiting the cultural and religious perspectives in the findings. Additionally, this review includes only one qualitative study, so the understanding of individual perspectives is not fully represented. There is a need for further qualitative studies. Furthermore, non-statistical syntheses of quantitative intervention effects present challenges. This is due to the difficulty of describing results without being selective or emphasizing some findings over others (32). More specifically, there is concern that the conclusions of narrative synthesis may overemphasize certain selected results (61). In the narrative synthesis, the results per study were accepted at face value to some extent, meaning that some results may be more valid than others. Given these limitations, this review may not be applicable for practice, as it does not include strong methodological studies (e.g., large national or international cohort studies). However, this review focuses on an underreported research issue, aiming to identify the risk factors of bereavement-related problems among refugees within the psycho-socio-cultural context of personhood and their needs. The findings indicate that aspects of personhood are significant for bereaved refugees and their formal and informal caregivers. To drive change, evidence should be presented to policymakers, public health authorities, healthcare providers, and refugee organizations to inform the development of culturally sensitive, person-centered care models. This issue should be framed as a public health priority, calling for a cohesive response that integrates bereavement care into refugee health services, ensuring adequate support to address the psychological needs of refugees and reduce long-term public health burdens. This review provides evidence of the need for further research focused on culturally sensitive, comprehensive psychosocial interventions related to aspects of personhood. Additionally, this study is significant as the first review on bereavement support for refugees in the psycho-socio-cultural context of personhood, which can help address the complex nature of bereavement and ensure high-quality care through the implementation of person-centered care.

## 5 Conclusion

This study highlights the need for psycho-socio-cultural bereavement support interventions and improved diagnostic tools for the early detection of grief symptoms, recognizing bereavement as a critical public health issue. To create a pathway for impact, policymakers, public health authorities, healthcare providers, and refugee support organizations must access this evidence to integrate bereavement care into public health frameworks, ensuring timely and effective interventions to reduce the broader societal burden. The results indicate that ring theory can be a useful tool for developing evidence-based practices that involve a cultural component specific to bereaved refugees from diverse backgrounds. Understanding the personhood of bereaved refugees in the context of their support needs and risk factors for poor bereavement outcomes is important to provide dignified, person-centered care and to develop a personalized support system. This review offers evidence to support the development of policy and practice involving culturally sensitive and more comprehensive bereavement interventions for bereaved refugees from diverse backgrounds. Findings from this systematic rapid review can also facilitate the construction of a standardized tool for the early detection of grief symptoms based on the psycho-socio-cultural concept of personhood for use by healthcare professionals. Moreover, this review may contribute to the establishment and improvement of support programs for prolonged grief disorder by providing healthcare professionals with a comprehensive and holistic perspective. Further studies are needed that focus on the cross-cultural measurement invariance of assessment tools and compare core symptomatology with potential additional culture-specific risk factors. Additionally, there is a need for more individual perspectives from bereaved refugees to understand the impact of individuals’ psycho-socio-cultural backgrounds on bereavement care.

## Data Availability

The original contributions presented in the study are included in the article/Supplementary material, further inquiries can be directed to the corresponding author.

## References

[1] UNHCR. (2023) Global forced displacement in 2023. Available online at: https://www.unhcr.org/refugee-statistics (Accessed December 5, 2023).

[2] Kokou-Kpolou CK Moukouta CS Masson J Bernoussi A Cénat JM Bacqué M-F. Correlates of grief-related disorders and mental health outcomes among adult refugees exposed to trauma and bereavement: a systematic review and future research directions. J Affect Disord. (2020) 267:171–84. doi: 10.1016/j.jad.2020.02.026, PMID: 32217217

[3] Parkes CM. Bereavement In: Parkes CM, editor. Social problems and mental health. New York: Routledge (2022). 19–21.

[4] Lichtenthal WG Roberts KE Donovan LA Breen LJ Aoun SM Connor SR . Investing in bereavement care as a public health priority. Lancet Public Health. (2024) 9:e270–4. doi: 10.1016/S2468-2667(24)00030-6, PMID: 38492580 PMC11110717

[5] Frounfelker RL Mishra T Dhesi S Gautam B Adhikari N Betancourt TS. “We are all under the same roof”: coping and meaning-making among older Bhutanese with a refugee life experience. Soc Sci Med. (2020) 264:113311. doi: 10.1016/j.socscimed.2020.113311, PMID: 32890976 PMC9412077

[6] Chan KJ Young MY Sharif N. Well-being after trauma: a review of posttraumatic growth among refugees. Can Psychol. (2016) 57:291–9. doi: 10.1037/cap0000065

[7] Xu Q. How resilient a refugee community could be: the Vietnamese of New Orleans. Traumatology. (2017) 23:56–67. doi: 10.1037/trm0000091

[8] Madi F Ismail H Fouad FM Kerbage H Zaman S Jayawickrama J . Death, dying, and end-of-life experiences among refugees: a scoping review. J Palliat Care. (2019) 34:139–44. doi: 10.1177/0825859718812770, PMID: 30458699

[9] Tay AK Mohsin M Rees S Tam N Kareth M Silove D. The structure and psychosocial correlates of complicated bereavement amongst refugees from West Papua. Soc Psychiatry Psychiatr Epidemiol. (2019) 54:771–80. doi: 10.1007/s00127-019-01666-1, PMID: 30778622

[10] Siriwardhana C Ali SS Roberts B Stewart R. A systematic review of resilience and mental health outcomes of conflict-driven adult forced migrants. Confl Heal. (2014) 8:1–14. doi: 10.1186/1752-1505-8-13, PMID: 25177360 PMC4149800

[11] Boelen PA Smid GE. Disturbed grief: prolonged grief disorder and persistent complex bereavement disorder. BMJ. (2017) 357:j2016. doi: 10.1136/bmj.j2016, PMID: 28522468

[12] Steil R Gutermann J Harrison O Starck A Schwartzkopff L Schouler-Ocak M . Prevalence of prolonged grief disorder in a sample of female refugees. BMC Psychiatry. (2019) 19:1–10. doi: 10.1186/s12888-019-2136-1, PMID: 31088419 PMC6518607

[13] Nickerson A Liddell BJ Maccallum F Steel Z Silove D Bryant RA. Posttraumatic stress disorder and prolonged grief in refugees exposed to trauma and loss. BMC Psychiatry. (2014) 14:1–11. doi: 10.1186/1471-244X-14-106, PMID: 24712883 PMC3998219

[14] Maciejewski PK Maercker A Boelen PA Prigerson HG. “Prolonged grief disorder” and “persistent complex bereavement disorder”, but not “complicated grief”, are one and the same diagnostic entity: an analysis of data from the Yale bereavement study. World Psychiatry. (2016) 15:266–75. doi: 10.1002/wps.20348, PMID: 27717273 PMC5032512

[15] Im H Neff J. Spiral loss of culture: cultural trauma and bereavement of Bhutanese refugee elders. J Immigr Refug Stud. (2021) 19:99–113. doi: 10.1080/15562948.2020.1736362

[16] Prigerson HG Boelen PA Xu J Smith KV Maciejewski PK. Validation of the new DSM-5-TR criteria for prolonged grief disorder and the PG-13-revised (PG-13-R) scale. World Psychiatry. (2021) 20:96–106. doi: 10.1002/wps.20823, PMID: 33432758 PMC7801836

[17] WHO. International classification of diseases for mortality and morbidity statistics. Geneva: WHO (2018).

[18] Killikelly C Bauer S Maercker A. The assessment of grief in refugees and post-conflict survivors: a narrative review of etic and emic research. Front Psychol. (2018) 9:1957. doi: 10.3389/fpsyg.2018.01957, PMID: 30405474 PMC6204364

[19] Lundorff M Holmgren H Zachariae R Farver-Vestergaard I O’Connor M. Prevalence of prolonged grief disorder in adult bereavement: a systematic review and meta-analysis. J Affect Disord. (2017) 212:138–49. doi: 10.1016/j.jad.2017.01.030, PMID: 28167398

[20] WHO. (2023) WHO report on refugee and migrant. Available online at: https://www.who.int/europe/health-topics/refugee-and-migrant-health#tab=tab_3 (Accessed December 2, 2023).

[21] Kpanake L. Cultural concepts of the person and mental health in Africa. Transcult Psychiatry. (2018) 55:198–218. doi: 10.1177/1363461517749435 29400136

[22] Radha Krishna LK Alsuwaigh R. Understanding the fluid nature of personhood–the R ing T heory of P ersonhood. Bioethics. (2015) 29:171–81. doi: 10.1111/bioe.12085, PMID: 24547934

[23] Chan NPX Chia JL Ho CY Ngiam LXL Kuek JTY Ahmad Kamal NHB . Extending the ring theory of personhood to the care of dying patients in intensive care units. Asian Bioeth Rev. (2022) 14:71–86. doi: 10.1007/s41649-021-00192-0, PMID: 34691261 PMC8526529

[24] Krishna LKR. Accounting for personhood in palliative sedation: the ring theory of personhood. Med Humanit. (2014) 40:17–21. doi: 10.1136/medhum-2013-010368, PMID: 24072720

[25] Krishna LKR Kwek SY. The changing face of personhood at the end of life: the ring theory of personhood. Palliat Support Care. (2015) 13:1123–9. doi: 10.1017/S1478951514000686, PMID: 24991916

[26] Chua KZY Quah ELY Lim YX Goh CK Lim J Wan DWJ . A systematic scoping review on patients’ perceptions of dignity. BMC Palliat Care. (2022) 21:118. doi: 10.1186/s12904-022-01004-4, PMID: 35787278 PMC9251939

[27] Davies DJ. Dividual identity in grief theories, palliative and bereavement care. Palliat Care Soc Pract. (2020) 14:2632352420921867. doi: 10.1177/2632352420921867, PMID: 32685923 PMC7346685

[28] Tricco AC Antony J Zarin W Strifler L Ghassemi M Ivory J . A scoping review of rapid review methods. BMC Med. (2015) 13:1–15. doi: 10.1186/s12916-015-0465-6, PMID: 26377409 PMC4574114

[29] Page MJ McKenzie JE Bossuyt PM Boutron I Hoffmann TC Mulrow CD . The PRISMA 2020 statement: an updated guideline for reporting systematic reviews. BMJ. (2021) 372:n171. doi: 10.1136/bmj.n71, PMID: 33782057 PMC8005924

[30] Open Grey. (2020) Grey literature in Europe. Available online at: https://opengrey.eu/ (Accessed December 1, 2023).

[31] End Note T. End note. End note X9 ed. Philadelphia, PA: Clarivate (2013).

[32] Higgins J. Cochrane handbook for systematic reviews of interventions. Hoboken, NJ: John Wiley & Son (2020).

[33] Popay J Roberts H Sowden A Petticrew M Arai L Rodgers M . Guidance on the conduct of narrative synthesis in systematic reviews final report. J Epidemiol Community Health. (2006) 1:b92. doi: 10.13140/2.1.1018.4643

[34] Hawker S Payne S Kerr C Hardey M Powell J. Appraising the evidence: reviewing disparate data systematically. Qual Health Res. (2002) 12:1284–99. doi: 10.1177/1049732302238251, PMID: 12448672

[35] Hinton DE Peou S Joshi S Nickerson A Simon NM. Normal grief and complicated bereavement among traumatized Cambodian refugees: cultural context and the central role of dreams of the dead. Cult Med Psychiatry. (2013) 37:427–64. doi: 10.1007/s11013-013-9324-0, PMID: 23868080 PMC3759644

[36] Bryant R Edwards B Creamer M O'Donnell M Forbes D Felmingham K . A population study of prolonged grief in refugees. Epidemiol Psychiatr Sci. (2020) 29:e44. doi: 10.1017/S2045796019000386, PMID: 31423962 PMC8061287

[37] Bryant RA Edwards B Creamer M O’Donnell M Forbes D Felmingham KL . Prolonged grief in refugees, parenting behaviour and children’s mental health. Aust N Z J Psychiatry. (2021) 55:863–73. doi: 10.1177/0004867420967420, PMID: 33124446

[38] Craig CD Sossou M-A Schnak M Essex H. Complicated grief and its relationship to mental health and well-being among Bosnian refugees after resettlement in the United States: implications for practice, policy, and research. Traumatology. (2008) 14:103–15. doi: 10.1177/1534765608322129

[39] Killikelly C Ramp M Maercker A. Prolonged grief disorder in refugees from Syria: qualitative analysis of culturally relevant symptoms and implications for ICD-11. Ment Health Relig Cult. (2021) 24:62–79. doi: 10.1080/13674676.2020.1825361

[40] Comtesse H Rosner R. Prolonged grief disorder among asylum seekers in Germany: the influence of losses and residence status. Eur J Psychotraumatol. (2019) 10:1591330. doi: 10.1080/20008198.2019.1591330, PMID: 30988893 PMC6450486

[41] Hinton DE Nickerson A Bryant RA. Prolonged grief in Cambodian refugees following genocide: rebirth concerns and avoidance of reminders. J Loss Trauma. (2013) 18:444–60. doi: 10.1080/15325024.2012.714218

[42] Kokou-Kpolou K Mbassa Menick D Moukouta CS Baugnet L Kpelly DE. A cross-cultural approach to complicated grief reactions among Togo–Western African immigrants in Europe. J Cross-Cult Psychol. (2017) 48:1247–62. doi: 10.1177/0022022117721972

[43] Tay AK Rees S Tam N Kareth M Silove D. Defining a combined constellation of complicated bereavement and PTSD and the psychosocial correlates associated with the pattern amongst refugees from West Papua. Psychol Med. (2019) 49:1481–9. doi: 10.1017/S0033291718002027, PMID: 30149819

[44] Hsu C-MK Kleim B Nicholson EL Zuj DV Cushing PJ Gray KE . Sex differences in intrusive memories following trauma. PLoS One. (2018) 13:e0208575. doi: 10.1371/journal.pone.0208575, PMID: 30521618 PMC6283557

[45] Smid GE Groen S de la Rie SM Kooper S Boelen PA. Toward cultural assessment of grief and grief-related psychopathology. Psychiatr Serv. (2018) 69:1050–2. doi: 10.1176/appi.ps.201700422, PMID: 30041592

[46] Kim J Tol WA Shrestha A Kafle HM Rayamajhi R Luitel NP . Persistent complex bereavement disorder and culture: early and prolonged grief in Nepali widows. Psychiatry. (2017) 80:1–16. doi: 10.1080/00332747.2016.1213560, PMID: 28409713

[47] Alemi Q James S Cruz R Zepeda V Racadio M. Psychological distress in afghan refugees: a mixed-method systematic review. J Immigr Minor Health. (2014) 16:1247–61. doi: 10.1007/s10903-013-9861-1, PMID: 23784146 PMC3912229

[48] Hynie M. The social determinants of refugee mental health in the post-migration context: a critical review. Can J Psychiatry. (2018) 63:297–303. doi: 10.1177/0706743717746666, PMID: 29202665 PMC5912301

[49] Knipscheer JW Sleijpen M Mooren T Ter Heide FJJ Van der Aa N. Trauma exposure and refugee status as predictors of mental health outcomes in treatment-seeking refugees. BJPsych Bull. (2015) 39:178–82. doi: 10.1192/pb.bp.114.047951, PMID: 26755950 PMC4706143

[50] McLellan J. Religious responses to bereavement, grief, and loss among refugees. J Loss Trauma. (2015) 20:131–8. doi: 10.1080/15325024.2013.833807

[51] Douglas AR. Working with bereaved asylum-seekers and refugees. Bereave Care. (2010) 29:5–9. doi: 10.1080/02682621.2010.522371

[52] Guo P Alajarmeh S Alarja G Alrjoub W Al-Essa A Abusalem L . Compounded trauma: a qualitative study of the challenges for refugees living with advanced cancer. Palliat Med. (2021) 35:916–26. doi: 10.1177/02692163211000236, PMID: 33765877 PMC8114446

[53] George M. A theoretical understanding of refugee trauma. Clin Soc Work J. (2010) 38:379–87. doi: 10.1007/s10615-009-0252-y

[54] Killikelly C Merzhvynska M Zhou N Stelzer E-M Hyland P Rocha J . Examination of the new ICD-11 prolonged grief disorder guidelines across five international samples. Clin Psychol Eur. (2021) 3:4159. doi: 10.32872/cpe.4159, PMID: 36397782 PMC9667123

[55] Killikelly C Kagialis A Henneman S Coronado H Demanarig D Farahani H . Measurement and assessment of grief in a large international sample. J Affect Disord. (2023) 327:306–14. doi: 10.1016/j.jad.2023.01.095, PMID: 36736540

[56] Aeschlimann A Heim E Killikelly C Arafa M Maercker A. Culturally sensitive grief treatment and support: a scoping review. SSM-mental. Health. (2024) 5:100325. doi: 10.1016/j.ssmmh.2024.100325

[57] Heim E Kohrt BA. Cultural adaptation of scalable psychological interventions. Clin Psychol Eur. (2019) 1:1–22. doi: 10.32872/cpe.v1i4.37679

[58] Aeschlimann A Heim E Hoxha A Triantafyllidou V Killikelly C Haji F . Cultural adaptation of an internet-based self-help app for grieving Syrian refugees in Switzerland. BMC Public Health. (2024) 24:3048. doi: 10.1186/s12889-024-20507-8, PMID: 39501191 PMC11536621

[59] Slobodin O de Jong JT. Family interventions in traumatized immigrants and refugees: a systematic review. Transcult Psychiatry. (2015) 52:723–42. doi: 10.1177/1363461515588855, PMID: 26047828

[60] Tay AK Khat Mung H Badrudduza M Balasundaram S Fadil Azim D Arfah Zaini N . Psychosocial mechanisms of change in symptoms of persistent complex bereavement disorder amongst refugees from Myanmar over the course of integrative adapt therapy. Eur J Psychotraumatol. (2020) 11:1807170. doi: 10.1080/20008198.2020.1807170, PMID: 33062211 PMC7534324

[61] Campbell M Katikireddi SV Sowden A Thomson H. Lack of transparency in reporting narrative synthesis of quantitative data: a methodological assessment of systematic reviews. J Clin Epidemiol. (2019) 105:1–9. doi: 10.1016/j.jclinepi.2018.08.019, PMID: 30196129 PMC6327109

