# Supplementary material for: Risk factors for poor bereavement outcomes and the support needs of adult bereaved refugees within the psychosocial-cultural concept of personhood: a systematic review
Source: Front Public Health. 2025 May 1;13:1517237. doi: 10.3389/fpubh.2025.1517237 (PMC12078022; doi:10.3389/fpubh.2025.1517237)
Supplement: Supplementary file 1 [file Table_1.docx]

**Supplementary files**

**Supplementary File S1 – PRISMA Checklists**

1. **PRISMA 2020 Checklist**

| **Section and Topic** | **Item #** | **Checklist item** | **Location where item is reported** |
| --- | --- | --- | --- |
| **TITLE** | | |  |
| Title | 1 | Identify the report as a systematic review. | Title |
| **ABSTRACT** | | |  |
| Abstract | 2 | See the PRISMA 2020 for Abstracts checklist. | See section B |
| **INTRODUCTION** | | |  |
| Rationale | 3 | Describe the rationale for the review in the context of existing knowledge. | Introduction |
| Objectives | 4 | Provide an explicit statement of the objective(s) or question(s) the review addresses. | Aim and research questions |
| **METHODS** | | |  |
| Eligibility criteria | 5 | Specify the inclusion and exclusion criteria for the review and how studies were grouped for the syntheses. | Inclusion and exclusion criteria and Supplementary appendix 2 |
| Information sources | 6 | Specify all databases, registers, websites, organisations, reference lists and other sources searched or consulted to identify studies. Specify the date when each source was last searched or consulted. | Search strategy |
| Search strategy | 7 | Present the full search strategies for all databases, registers and websites, including any filters and limits used. | Supplementary appendix 3 |
| Selection process | 8 | Specify the methods used to decide whether a study met the inclusion criteria of the review, including how many reviewers screened each record and each report retrieved, whether they worked independently, and if applicable, details of automation tools used in the process. | Study selection |
| Data collection process | 9 | Specify the methods used to collect data from reports, including how many reviewers collected data from each report, whether they worked independently, any processes for obtaining or confirming data from study investigators, and if applicable, details of automation tools used in the process. | Data extraction |
| Data items | 10a | List and define all outcomes for which data were sought. Specify whether all results that were compatible with each outcome domain in each study were sought (e.g. for all measures, time points, analyses), and if not, the methods used to decide which results to collect. | Data extraction |
|  | 10b | List and define all other variables for which data were sought (e.g. participant and intervention characteristics, funding sources). Describe any assumptions made about any missing or unclear information. | Data extraction |
| Study risk of bias assessment | 11 | Specify the methods used to assess risk of bias in the included studies, including details of the tool(s) used, how many reviewers assessed each study and whether they worked independently, and if applicable, details of automation tools used in the process. | Quality assessment |
| Effect measures | 12 | Specify for each outcome the effect measure(s) (e.g. risk ratio, mean difference) used in the synthesis or presentation of results. | N/A |
| Synthesis methods | 13a | Describe the processes used to decide which studies were eligible for each synthesis (e.g. tabulating the study intervention characteristics and comparing against the planned groups for each synthesis (item #5)). | Data analysis |
|  | 13b | Describe any methods required to prepare the data for presentation or synthesis, such as handling of missing summary statistics, or data conversions. | N/A |
|  | 13c | Describe any methods used to tabulate or visually display results of individual studies and syntheses. | Data analysis |
|  | 13d | Describe any methods used to synthesize results and provide a rationale for the choice(s). If meta-analysis was performed, describe the model(s), method(s) to identify the presence and extent of statistical heterogeneity, and software package(s) used. | Data analysis |
|  | 13e | Describe any methods used to explore possible causes of heterogeneity among study results (e.g. subgroup analysis, meta-regression). | N/A |
|  | 13f | Describe any sensitivity analyses conducted to assess robustness of the synthesized results. | N/A |
| Reporting bias assessment | 14 | Describe any methods used to assess risk of bias due to missing results in a synthesis (arising from reporting biases). | N/A |
| Certainty assessment | 15 | Describe any methods used to assess certainty (or confidence) in the body of evidence for an outcome. | N/A |
| **RESULTS** | | |  |
| Study selection | 16a | Describe the results of the search and selection process, from the number of records identified in the search to the number of studies included in the review, ideally using a flow diagram. | Included studies and Figure 2 |
|  | 16b | Cite studies that might appear to meet the inclusion criteria, but which were excluded, and explain why they were excluded. | Figure 2 |
| Study characteristics | 17 | Cite each included study and present its characteristics. | Supplementary appendix 5 and included studies |
| Risk of bias in studies | 18 | Present assessments of risk of bias for each included study. | Results quality assessment and supplementary appendix 4 |
| Results of individual studies | 19 | For all outcomes, present, for each study: (a) summary statistics for each group (where appropriate) and (b) an effect estimate and its precision (e.g. confidence/credible interval), ideally using structured tables or plots. | Table 1 and Supplementary appendix 5 |
| Results of syntheses | 20a | For each synthesis, briefly summarise the characteristics and risk of bias among contributing studies. | Table 2 and reults |
|  | 20b | Present results of all statistical syntheses conducted. If meta-analysis was done, present for each the summary estimate and its precision (e.g. confidence/credible interval) and measures of statistical heterogeneity. If comparing groups, describe the direction of the effect. | N/A |
|  | 20c | Present results of all investigations of possible causes of heterogeneity among study results. | N/A |
|  | 20d | Present results of all sensitivity analyses conducted to assess the robustness of the synthesized results. | N/A |
| Reporting biases | 21 | Present assessments of risk of bias due to missing results (arising from reporting biases) for each synthesis assessed. | N/A |
| Certainty of evidence | 22 | Present assessments of certainty (or confidence) in the body of evidence for each outcome assessed. | N/A |
| **DISCUSSION** | | |  |
| Discussion | 23a | Provide a general interpretation of the results in the context of other evidence. | Discussion |
|  | 23b | Discuss any limitations of the evidence included in the review. | Discussion |
|  | 23c | Discuss any limitations of the review processes used. | Discussion |
|  | 23d | Discuss implications of the results for practice, policy, and future research. | Discussion |
| **OTHER INFORMATION** | | |  |
| Registration and protocol | 24a | Provide registration information for the review, including register name and registration number, or state that the review was not registered. | Abstract and methods |
|  | 24b | Indicate where the review protocol can be accessed, or state that a protocol was not prepared. | Abstract and methods |
|  | 24c | Describe and explain any amendments to information provided at registration or in the protocol. | N/A |
| Support | 25 | Describe sources of financial or non-financial support for the review, and the role of the funders or sponsors in the review. | Author contributions |
| Competing interests | 26 | Declare any competing interests of review authors. | Declaration of interest statement |
| Availability of data, code and other materials | 27 | Report which of the following are publicly available and where they can be found: template data collection forms; data extracted from included studies; data used for all analyses; analytic code; any other materials used in the review. | Funding resources |

*From:*  Page MJ, McKenzie JE, Bossuyt PM, Boutron I, Hoffmann TC, Mulrow CD, et al. The PRISMA 2020 statement: an updated guideline for reporting systematic reviews. BMJ 2021;372:n71. doi: 10.1136/bmj.n71

1. **PRISMA 2020 for Abstracts Checklist**

| **Section and Topic** | **Item #** | **Checklist item** | **Reported (Yes/No)** |
| --- | --- | --- | --- |
| **TITLE** | | |  |
| Title | 1 | Identify the report as a systematic review. | Yes |
| **BACKGROUND** | | |  |
| Objectives | 2 | Provide an explicit statement of the main objective(s) or question(s) the review addresses. | Yes |
| **METHODS** | | |  |
| Eligibility criteria | 3 | Specify the inclusion and exclusion criteria for the review. |  |
| Information sources | 4 | Specify the information sources (e.g. databases, registers) used to identify studies and the date when each was last searched. | Yes |
| Risk of bias | 5 | Specify the methods used to assess risk of bias in the included studies. | Yes |
| Synthesis of results | 6 | Specify the methods used to present and synthesise results. | Yes |
| **RESULTS** | | |  |
| Included studies | 7 | Give the total number of included studies and participants and summarise relevant characteristics of studies. | Yes |
| Synthesis of results | 8 | Present results for main outcomes, preferably indicating the number of included studies and participants for each. If meta-analysis was done, report the summary estimate and confidence/credible interval. If comparing groups, indicate the direction of the effect (i.e. which group is favoured). | Yes |
| **DISCUSSION** | | |  |
| Limitations of evidence | 9 | Provide a brief summary of the limitations of the evidence included in the review (e.g. study risk of bias, inconsistency and imprecision). | No |
| Interpretation | 10 | Provide a general interpretation of the results and important implications. | Yes |
| **OTHER** | | |  |
| Funding | 11 | Specify the primary source of funding for the review. | No |
| Registration | 12 | Provide the register name and registration number. | Yes |

*From:*  Page MJ, McKenzie JE, Bossuyt PM, Boutron I, Hoffmann TC, Mulrow CD, et al. The PRISMA 2020 statement: an updated guideline for reporting systematic reviews. BMJ 2021;372:n71. doi: 10.1136/bmj.n71

**Supplementary File S2. Eligibility criteria**

| **Inclusion Criteria** | **Exclusion Criteria** |
| --- | --- |
| **Sample**   - Adult refugees ≥18 years - Refugees who had experienced the death of a family member/friend - Any mixed population including the bereavement adult refugees   **Context**   - Bereavement care   **Study design**   - Qualitative - Quantitative - Mixed methods - Primary and secondary analysis of data   **Outcomes**   - Risk factors in bereavement outcome - Bereavement support needs | **Sample**   - Individuals <18 years old - Not bereaved refugee population   **Context**   - Not in the context of bereavement care of refugees   **Study design**   - Case studies, Systematic reviews, Unpublished studies/conference papers/editorial papers.   **Outcomes**   - Not included refugees’ bereavement support needs and the risk factors in bereavement outcome. |
| **Language**   - English language | **Language**   - Non-English language |
| **Date and country**   - We will not apply any geographic limits or publication date restrictions |  |

**Supplementary File S3. Example Search Strategy for MEDLINE**

| **Database** | | | **Keywords** | **Results** |
| --- | --- | --- | --- | --- |
| **MEDLINE (EBSCOhost**) | S1 | AB ("bereavement care" or "bereavement support" or "palliat*" OR "terminal*" OR "end of life care" OR "terminal care" OR "comfort care" OR "hospice*" OR "end of life care" OR "dying" OR "dying experience" OR "end of life") OR TI ("bereavement care" or "bereavement support" or "palliat*" OR "terminal*" OR "end of life care" OR "terminal care" OR "comfort care" OR "hospice*" OR "end of life care" OR "dying" OR "dying experience" OR "end of life") | | 656,144 |
|  | S2 | (MH "Palliative Medicine") OR (MH "Palliative Care") OR (MH "Hospice and Palliative Care Nursing") OR (MH "Terminal Care") OR (MH "Hospice Care") | | 93,351 |
|  | S3 | TI (( griev*OR mourn* OR reaction* OR bereave* OR grief*) ) OR AB ( ( griev*OR mourn* OR reaction* OR bereave* OR grief* ) | | 2,090,767 |
|  | S4 | (MH "Bereavement") OR (MH "Grief") OR ("Prolonged grief disorder") | | 15,781 |
|  | S5 | AB (refugee* OR migrant* OR immigrant* OR emigrant* OR migration* OR emigration* OR immigration* OR inmigration* OR outmigration* OR asylum seeker* OR asylee* OR "incomer*" OR "in comer*" OR "new comer*" OR newcomer* OR migrant* OR resettler* OR displaced person* OR displaced people ) OR TI (refugee* OR migrant* OR immigrant* OR emigrant* OR migration* OR emigration* OR immigration* OR inmigration* OR outmigration* OR asylum seeker* OR asylee* OR "incomer*" OR "in comer*" OR "new comer*" OR newcomer* OR migrant* OR resettler* OR displaced person* OR displaced people) | | 410,722 |
|  | S6 | (MH "Emigrants and Immigrants") OR (MH "Refugees") OR (MH "Emigration and Immigration") OR (MH "Undocumented Immigrants") OR (MH "Human Migration") OR (MH "Transients and Migrants") | | 64,328 |
|  | S7 | AB (need* or demand* or requirement* or risk factor* or perception* or attitude* or value* or view* or perspective* or experience* or symptom*OR TI (need* or demand* or requirement* or risk factor* or perception* or attitude* or value* or view* or perspective* or experience*or symptom*) | | 8,830,365 |
|  | S8 | (MH "risk factors") OR MH ("Needs Assessment") (MH "Attitude") OR (MH "Perception") OR (MH "Value of Life") OR (OR (MH "Attitude to Death") | | 1,028,557 |
|  | S9 | S1 OR S2 | | 683,084 |
|  | S10 | S3 OR S4 | | 2,090,524 |
|  | S11 | S5 OR S6 | | 429,161 |
|  | S12 | S7 OR S8 | | 8,830,365 |
|  | **S13** | **S9 AND S10 S11 AND S12** | | **106** |

**Supplementary Files S4. Quality assessment**

Summary of the included studies’ critical appraisal using the appraisal tool by Hawker et al. (2002)

| **Study** | **Abstract/**  **title** | **Introduction/aims** | **Method/**  **data** | **Sampling** | **Data analysis** | **Ethics** | **Bias** | **Findings/**  **results** | **Transferability/**  **generalisability** | **Implications &**  **usefulness** | **Total** |
| --- | --- | --- | --- | --- | --- | --- | --- | --- | --- | --- | --- |
| **Bryant et al. (2019)** | 4 | 4 | 4 | 4 | 4 | 3 | 3 | 4 | 4 | 3 | 37 |
| **Bryant et al. (2021)** | 4 | 4 | 4 | 4 | 4 | 1 | 1 | 4 | 4 | 3 | 33 |
| **Comtesse & Rosner (2019)** | 4 | 4 | 3 | 3 | 4 | 1 | 1 | 4 | 3 | 3 | 30 |
| **Craig et al. (2008)** | 3 | 4 | 4 | 3 | 4 | 1 | 1 | 4 | 3 | 4 | 31 |
| **Hinton et al (2013a)** | 3 | 4 | 3 | 2 | 3 | 1 | 1 | 3 | 2 | 2 | 24 |
| **Hinton et al (2013b)** | 3 | 3 | 2 | 2 | 2 | 1 | 1 | 2 | 2 | 2 | 20 |
| **Killikelly et al. (2021)** | 4 | 4 | 4 | 3 | 4 | 3 | 3 | 4 | 2 | 3 | 34 |
| **Kokou-Kpolou et al. (2017)** | 4 | 4 | 4 | 2 | 4 | 4 | 3 | 4 | 3 | 4 | 36 |
| **Nickerson et al. (2014)** | 4 | 4 | 4 | 3 | 4 | 4 | 4 | 4 | 3 | 4 | 38 |
| **Steil et al. (2019)** | 4 | 4 | 4 | 4 | 4 | 4 | 4 | 4 | 2 | 3 | 37 |
| **Tay et al. (2018)** | 4 | 4 | 4 | 4 | 4 | 3 | 3 | 4 | 3 | 4 | 37 |
| **Tay et al. (2019)** | 4 | 4 | 3 | 3 | 4 | 3 | 3 | 4 | 3 | 4 | 35 |

**Scoring items:** 4 = good; 3 = fair; 2 = poor; 1 = very poor

**Scoring total:** good = 40–31; fair = 30–21; poor = 20–11; very poor = 10-1

**Supplementary File S5. Refugees’ bereavement care: Data extraction of results (n=11)**

| **Author (Year)**  **Country** | **Aim** | **Design/**  **method** | **Population/ Bereavement-related characteristics** | **Bereavement support needs** | **Risk factors including innate, individual, relational and societal** |
| --- | --- | --- | --- | --- | --- |
| **Kokou-Kpolou et al.(2017)**  **France** | To examine how migration trajectory (including immigration status and length of time) and the utilisation of ritual practices to manage feelings of grief are connected within the context of migration. | Survey study | N=74 (54 migrants and 20 refugees) in France and Belgium  - The mean age of the refugees: 45.9 years.  -62.2 % men and 37.8 % women  -Duration of immigration : More than 10 years: 56.8%  -Duration of bereavement: More than 3 years: 48.6%  -The prevalence of prolonged grief disorder: 41.9%. | -Bereavement rituals on the process of adapting to the loss of a loved one are essential to cope with the death of close one.  - Heuristic understanding of the effects of bereavement following the death of a parent or close relative according to the individual’s migration trajectory and the usefulness of ritual and social support to mitigate the anguish of death across time and space.  -Focusing the psycho-cultural aspects of the grief reactions | **Innate:**  -Fewer grief complications tended to occur with deaths anticipated due to old age, long-term illness, or chronic disease compared to unexpected deaths.  -The eldest siblings showed a notably high occurrence of complicated grief.  **Individual:**  -Feeling guilty, dazed or stunned, loneliness, bitterness, numbness, and emptiness made up the spectrum of severe and persistent guilt reactions.  -Viewing the deceased's body and bidding a final farewell through ritualistic gestures alleviate distress and initiate mental processes crucial for accepting the loss. Individuals engaging in these bereavement rituals experienced reduced feelings of guilt and despair.  **Relational:**  -The prominence of feelings of loneliness and nostalgia, which seem to denote, by association, the consolidation of memory traces of the migration experience.  -Grief reactions developed essentially around the kinship tie (and indirectly the parent–child relationship). Kinship tie: Father 19 (Johnston & Narayanasamy); Mother 15 (20.3%); Grandfather/mother 18 (24.3%); Uncle, aunt 13 (17.6%); Brother, sister 9 (12.2%)  -Grief reactions develop based on the parent–child relationship inextricably associated with a feeling of belonging to the ethnic group and collective memory.  **Societal:**  -Refugees experience greater difficulty in achieving social and professional integration. A majority of refugees reported a deterioration of their social life when the duration of their immigration exceeded 10 years.  -In cases where the death took place in the native country, administrative red tape and/or financial problems were the main reasons invoked to justify not participating in the bereavement rituals.  -Findings indicate that the political, administrative, and economic constraints in the context of migration amplify the experience of bereavement. |
| **Nickerson et al.**  **(2014)**  **Australia** | To (1) determine whether there are distinct classes of PTSD and PGD symptoms in bereaved trauma survivors exposed to conflict and persecution, and (2) examine whether particular types of refugee experiences and stressors differentially predict symptom profiles. | Survey study using latent class analysis | 248 Mandaean adult refugees in Australia following persecution in Iraq.  -48% male, 52% women. | - Maintaining cultural traditions  -Identifying specific symptom profiles in individuals exposed to both trauma and loss.   -The development of intervention strategies that target specific types of distress in survivors of persecution and conflict. - Access to counselling services | **Innate:**  - Higher levels of depression symptoms compared to the resilient class; combined PGD  - Female, to have been exposed to more types of detention/abuse traumas and traumatic losses, and to report greater difficulties associated with loss of culture and support.  -Compared to those in the resilient class, those in the PGD class were more likely to be older, to have reported exposure to more types of detention/abuse trauma and to have experienced greater adaptation difficulties.  **Individual:** N/A  **Relational:** N/A  **Societal:** N/A |
| **Bryant et al. (2019)**  **Australia** | To determine the prevalence of probable PGD and its associated problems. | Prospective cohort study of refugees admitted to Australia | 1245 refugees (Bhutan or Myanmar (213, 17.1%), Iran (100, 8.1%), Libya/Syria/Egypt (86, 6.9%), Pakistan (76, 6.1%), sub-Sahara Africa and Sri Lanka/India (35, 2.8%).  -The majority of adult participants were male (785, 61.7%) | -Psychological assistance need (Only 56.3% of those with probable PGD reported ever receiving psychological assistance in Australia -but of those, 91.4% did so in the last 12 months-, compared to 35.0% of those without PGD)  -Help-seeking among people with PGD is often low, with one study finding that only 43% of those with PGD sought mental health assistance. The need to overcome the apparent barriers for refugees with PGD to access mental health services.  - The low rate of access to mental health assistance for these refugees highlights that there is a need to address this issue in refugee populations.  -Sub-optimal help-seeking in bereaved refugees may be due to stigma about attending mental health services, ignorance of appropriate referral opportunities or avoidance of confronting emotional discomfort associated with their grief  -In the context of strong evidence for the efficacy of grief-focused therapies in alleviating PGD symptoms there are good reasons to provide more targeted programmes to address the persistent grief problems experienced by refugees. | **Innate:**  -Refugees with probable PGD were more likely to be female.  -In terms of problems associated with persistent grief, those with probable PGD were more likely to be older, and to report severe mental illness, probable PTSD, and disability.  **Relational:**  -Refugees with probable PGD were more likely to be not married.  **Individual:** N/A  **Societal:**   -Concerns about refugees adjusting to their new lives in the host country is related to persistent grief problems are associated with compounded psychological and social challenges  -In terms of problems associated with persistent grief, those with probable PGD were more likely to be unemployed and difficulty to trust others in the community.  -Refugees were markedly more likely to develop probable PGD if they had suffered murder or dis- appearance of a family member (32.8  Murder/disappearance of family)  -Refugees with probable PGD were more likely to have experienced discrimination |
| **Comtesse, H & Rosner, R (2019)**  **Germany** | To investigate the rate and potential risk factors of PGD in recently fled asylum seekers who lived in collective accommodations in Germany. | Questionnaire-based semi-structured clinical interviews | Asylum seekers from different countries (N = 99)  Participants were mostly male. The majority of participants had Arabic (45%), Kurdish, and Afghan (15%) backgrounds.  - The total sample was on average 30.12 years old (range: 19–74). | Problematic grief needs to be considered in health care policies for the current refugee population in Europe.  -The need to screen for problematic grief in the current refugee population in Europe.  -Although screening instruments for the most common mental disorders have been recently validated among asylum seekers , a short prolonged grief screening is lacking.  -The inclusion of a grief screening in the initial medical examination after arrival might be helpful to refer asylum seekers to more extensive evaluations. Future work on refugees should examine the long-term development of grief trajectories.  -The development of grief-specific interventions for asylum seekers might be needed not only to improve psychological functioning but also to foster social and economic integration into the host society | **Innate:**  -Residence status, symptoms of depression, and PTSD symptoms were entered in the model, accounting for additional 25% of the variance.  **Individual:** N/A  **Relational:**  -64% loss of a nuclear family member, 89% loss of an extended family member, 88% loss of a friend.  The number of lost nuclear family members emerged as a positive predictor of PGD symptoms  **Societal:**  -Prolonged social isolation, unemployment, persistent bad living conditions and residence permit were all significantly associated with PGD symptoms.  -All participants had experienced at least one event that would be considered as potentially traumatic according to DSM-5.  -Severe human suffering (74%), transportation accidents (73%), and combat or war-zone exposure (69%) were the most frequently reported events by all participants.  -Physical assault: 51.5 %  -Assault with a weapon: 46.4 %  Life-threatening illness or injury: 55.5 % Combat or exposure to war-zone : 69.7 % |
| **Steil et al (2019)**  **Germany** | To examine the prevalence of PGD in female refugees in Germany according to the criteria proposed by Prigerson and colleagues in 2009, and to associate PGD with other common psychopathology (e.g. anxiety, depression, somatization and trauma). | Cross-sectional study | -106 adult female refugees  -Mean age of participants: 29.26years (SD=8.75).  -90 of the 106 participants had experienced bereavement, and among those, 9.41% experience PGD  The majority of the participants came from Afghanistan (36.14%, n = 30) and Syria (32.53%, n = 27), followed by Iran (10.84%, n = 9), Iraq (9.64%, n = 8), Eritrea (6.02%, n = 5) and Somalia (2.41%, n = 2). 2.41% (n = 2) had other citizenships (Tajik, Turkmen)  -76.47% of participants are Muslims. | -The need for assessment and specifically tailored treatment of PGD in refugees. PGD goes along with significant psychopathology, which further emphasizes the need for treatment.  PGD should be considered when assessing psychological disorders among this group and when planning interventions.  - Tools for diagnostic assessment either have to be newly developed in order to be sensitive towards cultural peculiarities or have to be adapted at least. The same applies for the dissemination of specific interventions.  - Standard grief measures, which have been validated by Western non-refugee populations mostly, do not assess these kinds of symptoms, which makes it even more important to assess them at least separately  - Cultures have very different norms when it comes to mourning, suggesting that mourning for a longer period of time may be considered acceptable and normal in one culture but not in another. Knowledge of the patient’s culture will benefit clinician-patient interaction and patient satisfaction in general, creating a major impact on mental health and well-being. | **Innate:**  -Grief symptoms were significantly associated with symptoms of depression, anxiety, somatization.  Individual:  -Family status and religion were significant socio-demographic predictors of PGD.  **Relational:**  -72.94% were married and living together All individuals suffering from PGD.  -There was a trend for having children to also be associated with PGD.  **Societal:**  -Grief symptoms were significantly associated with the number of experienced traumatic events |
| **Bryant et al. (2021)**  **Australia** | To which prolonged grief disorder in refugees is associated with their parenting behaviour and in turn with their children’s mental health. | Prospective cohort study of refugees | 110 adults  -37% of bereaved refugees experience PGD  -71.8% Female; 28.2% Male  --The majority of these partici- pants came from Iraq or Afghanistan (93, 84.5%); and the remainder chiefly from Bhutan or Myanmar (6, 5.5%), Iran (5, 4.5%) and Sub-Saharan Africa (5, 4.5%). /110) | -The association between parenting style, grief severity and children’s mental health highlights that managing grief reactions in refugees can benefit both refugees and their children.  The risk that PGD in refugees poses for the mental health of their children should be considered.  - Severity of prolonged grief disorder is directly linked to refugee children’s mental health.  -Caregivers’ grief was directly associated with children’s emotional difficulties. More harsh parenting was associated with children’s conduct problems, and this was most apparent in caregivers with less severe grief.  - More harsh parenting was associated with children’s conduct problems, and this was more evident in those with less severe grief. | **Innate:**  -There were significant paths from female adult gender and older age to PGD severity, and there were significant indirect paths to emotional difficulties: from age and from female gender  **Individual:** N/A  **Relational:**  -Caregivers’ grief was directly associated with children’s emotional difficulties.  **Societal:** N/A |
| **Tay et al.(2019)**  **Australia** | To investigate the manifestations, prevalence, factorial structure and psychosocial correlates of complicated bereavement amongst refugees from West Papua, a population with no past exposure to western concepts of grief or to formal mental health services. | Mix method study using focus group and survey | 470 adults, 18 years and older born in West Papua  21% met criteria for PGD based on ICD-11 criteria | -There is a pressing need to formulate and trial culturally congruent approaches to psychological interventions for complicated bereavement for refugees from a diversity of backgrounds. | **Innate:** N/A  **Individual:**  -In this study has wider implications for the nosology of mental disorders in suggesting that cultural, historical and contextual factors may influence the expression and emphasis given to symptoms across diagnostic categories.  -Culture and exposure to persecution and displacement contribute to the content and configuration of the complicated bereavement reaction  **Relational:** N/A  **Societal:**  -Traumatic losses, postmigration living difficulties, and the length of displacement as being associated with the overall symptoms of complicated bereavement |
| **Hinton et al. (2013)**  **USA** | To investigate whether prolonged grief (Prigerson et al.) forms a coherent syndrome that is discrete from posttraumatic stress disorder (PTSD) in a Cambodian refugee population and examine the relative centrality to PG of “avoidance of reminders of the deceased” as compared to “rebirth concerns” in the group. | Survey-based study | 100 individuals  -Mean age:54 (SD=7)  -46% (46 of 100) of the participants reported the loss of a parent; 29% said the loss of a sibling; 15% the loss of a child; 3% the loss of a husband; 3% the loss of an adoptive parent; 2% the loss of a friend; and 2% the loss of a grandparent.  -The mean time since the death was 28.9 years (SD = 11.9), and 69% of the index losses occurred during the Pol Pot period.  -According to the PG-14% (8 of 100) of the participants met criteria for a diagnosis of PGD. Fifty-nine percent of the participants reported grief-related functional impairment. The mean age of participants with PGD was 55 years (SD = 8), with 25% being male. The mean age of participants without PGD was 53 (SD = 7), with 34% being male. | -When examining the utility of diagnostic criteria in describing psychological phenomena within a specific group, it is important to consider the cultural, religious, and historical context in which the symptoms occur.  -Clinicians working with bereaved individuals from other cultural backgrounds should also consider the culturally specific beliefs and norms surrounding bereavement to more effectively understand and address grief reactions across cultural groups.  -In certain cultures concerns about the spiritual state of the deceased may be a core part of bereavement. It is incumbent on the clinician to determine the patient’s thoughts about the spiritual state of the deceased and cultural ideas about how “rebirth” or pas- sage to a more auspicious spiritual plane may be accomplished. | **Innate:**  -Loss experiences were inextricably intertwined with traumatic events.  - In the hierarchical linear regression predicting grief-related distress as assessed by the culturally sensitive measure (i.e., the CSM-G), the model evidenced adequate fit. PTSD symptoms, entered in the first step, significantly predicted grief-related distress (CSM-G).  -PGD symptoms entered in the second step, significantly predicted grief-related distress (CSM-G) over and above the variance explained by PTSD symptoms. The relationship between PTSD symptoms and grief-related distress (CSM-G) became nonsignificant when controlling for PGD symptoms. In the hierarchical logistic regression predicting grief-related functional impairment, a similar pattern emerged, with the relationship between PTSD and grief-related functional impairment becoming nonsignificant when control- ling for PGD symptoms.  **Individual:**  -The correlation analyses showed the rebirth concerns item to be very highly correlated to the two measures of bereavement severity (the one-item cultural measure and the PG-13), much more so than the avoidance of reminders item found in the PG-13.  -The two items that best predicted grief-related functional impairment were bitterness over the loss and concerns about rebirth status. These two items were often endorsed and had high sensitivity, and they also had high negative predictive power, meaning that in their absence grief-related impairment was unlikely.  **Relational:** N/A  **Societal:**  -If a severity score of 4 or 5 on this item (viz., whether the person has missed the deceased in the last month to the point of feeling poorly in the mind or body) is considered sufficient for caseness, then 31% of the sample met criteria for complicated bereavement. |
| **Tay et al. (2018)**  **Australia** | To investigate whether a combined pattern of complicated bereavement and PTSD can be identified amongst West Papuan refugees participating in an epidemiological survey  in a remote town in Papua New Guinea. | A survey study using latent class analysis | -486 adult West Papuans  -%20 PGD | -Needs support systems including safety and security; interpersonal bonds and networks; justice; roles and identities; and existential meaning.  -Needs cultural adapted interventions and more comprehensive psychological interventions. | **Innate:** N/A  **Individual:** N/A  **Relational:** N/A  **Societal:** N/A |
| **Craig et al (2008)**  **USA** | To investigate mental health and well-being variables in a randomly selected sample of Bosnian refugees. | Survey study | 500 Bosnian refugees with 126 surveys returned  Female with a mean age of 42. | -Mental health service providers should be properly educated regarding the barriers and should develop evidence-based practices that include a cultural component specific to the population of refugees being served.  -Recognition must be given to the multiple barriers that plague refugee populations with regard to the help-seeking process as a result of cultural beliefs that include shame and stigma associated with mental health and mental illness as well as language barriers.  -Focus is placed primarily on screening for communicable dis- eases; hence, significant opportunities to enhance the mental health of refugees have been missed.  -A policy change is needed, which also addresses the welfare of the refugee in terms of mental health. Addressing refugee mental health will possibly benefit the host society as well, and further research is needed in this area, especially in terms of more effective resettlement resulting in enhanced productivity, lower crime rates, and less cost to the medical system. | **Innate:**  -Correlational analysis was run to investigate the relationship of PGD to PTSD, anxiety, depression, well-being, and mental health.  -The mean difference was significant between women and men in regard to total complicated grief symptomatology with women scoring higher than men on the PGD  -Age differences were found for PGD with the oldest age cohort (≥55) having higher mean scores that were significantly different than the four younger age group’s mean scores .  **Individual:** N/A  **Relational:** N/A  **Societal:** N/A |
| **Killikelly et al. (2021)**  **Switzerland** | Two main aims: (1) to identify common symptoms of normal or abnormal grief in refugees from Syria (2) to identify barriers to the acceptability of the new prolonged grief disorder. | Exploratory qualitative study | 10 refugees from Syria  -Age ranged 25-28  -6 men and 4 women | -Need to mental health assessment measures.  -Need to conduct a brief interview with the client such as the Grief and Bereavement cultural interview  -Alternatives for mental health care offered. For example, care within the community or family may be prioritised. | **Innate:**  -The refugee experience aggravates grief symptoms  - In terms of the expectations for women, female gender is seen as a risk factor for severe grief. For example different expectations for emotional expression especially for men. A man is not supposed to show his emotions and feelings. He has to be strong and control himself.  -The same participant mentioned that a man could suffer from a stroke or diabetes because of the pain. Thus, a physical response could be one of the few signs that a man is suffering because of the death of a loved one.  -Themes related to the refugee experience are grouped into three different clusters: post-migration factors (residency status and adjustment difficulties), lack of connection with cultural identity and others (loss of homeland, lack of social network, inability to perform spiritual and religious practices), and ambiguous loss and uncertainty.  -Stigma - a negative perception of mental health services such as stigmatised as a crazy  **Individual:**  -Identified difficulties with adjusting to the culture in Switzerland.  -Loss of connection with culture and others (inability to perform spiritual and religious prac- tices, loss of homeland, lack of social network). he inability to engage in the traditional rituals of mourning were thought to aggravate the grief response.  -Mourning rituals  **Relational:**  -Some participants miss close people or do not have a social network.  -Ambiguous loss - uncertainty  **Societal:**  -The lack of access to health services or the uncertainty of having an F permit  -The loss of the homeland was another crucial factor. For example, one participant said that not being able to go back to Syria worsens grief |
